# Ultra-fast variant effect prediction using biophysical transcription factor binding models

**DOI:** 10.1093/nar/gkaf940

**Published:** 2025-10-08

**Authors:** Rezwan Hosseini, Ali Tugrul Balci, Dennis Kostka, Nathan Clark, Maria Chikina

**Affiliations:** Department of Computation and Systems Biology, University of Pittsburgh, Pittsburgh, PA 15213, United States; Genentech Inc., Computational Biology and Translation, South San Francisco, CA 94080, United States; Department of Computation and Systems Biology, University of Pittsburgh, Pittsburgh, PA 15213, United States; Department of Biological Science, University of Pittsburgh, Pittsburgh, PA 15260, United States; Department of Computation and Systems Biology, University of Pittsburgh, Pittsburgh, PA 15213, United States

## Abstract

Sequence variation within transcription factor (TF)-binding sites can significantly affect TF–DNA interactions, influencing gene expression and contributing to disease susceptibility or phenotypic traits. Despite recent progress in deep sequence-to-function models that predict functional output from sequence data, these methods perform inadequately on some variant effect prediction tasks, especially with common genetic variants. This limitation underscores the importance of leveraging biophysical models of TF binding to enhance interpretability of variant effect scores and facilitate mechanistic insights. We introduce *motifDiff*, a novel computational tool designed to quantify variant effects using mono- and dinucleotide position weight matrices. *motifDiff* offers several key advantages, including scalability to score millions of variants within minutes, implementation of statistically rigorous normalization strategy critical for optimal performance, and support for both dinucleotide and mononucleotide models. We demonstrate *motifDiff*’s efficacy by evaluating it across diverse ground truth datasets that quantify the effects of common variants *in vivo*, thereby establishing robust benchmarks for the predictive value of variant effect calculations. Finally, we show that our tool provides unique insights when interpreting human accelerated regions. *motifDiff* is available as a standalone Python application at https://github.com/rezwanhosseini/MotifDiff.

## Graphical Abstract

**Graphical Abstract:**
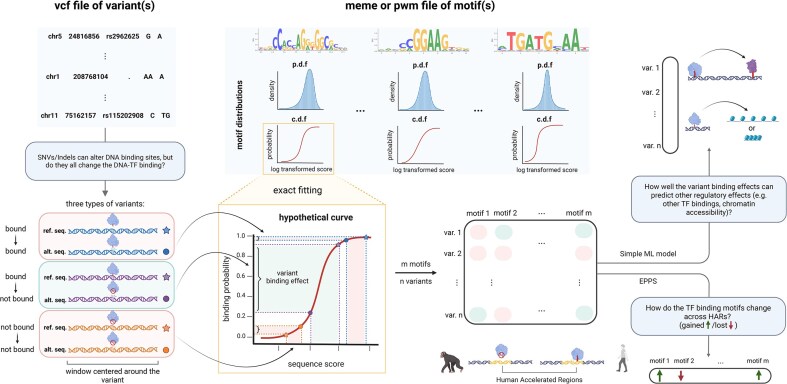

## Introduction

Understanding the functional consequences of genetic variants is a cornerstone of modern genomics and molecular biology. As our ability to identify genetic variation associated with disease and phenotypic traits continues to advance, so does the need for efficient tools to assess the potential impact of these variants on gene regulation. One crucial aspect of gene regulation is the binding of transcription factors (TFs) to specific DNA sequences. Variants within TF-binding sites (TFBSs) can disrupt or enhance TF–DNA interactions, thereby influencing gene expression and ultimately contributing to disease susceptibility or phenotypic traits.

In this context, a fast and reliable computational tool that can rapidly quantify the effect of genetic variants on TF binding is of critical importance. In recent years, noncoding variant interpretation has been driven by the use of deep sequence-to-function models [1–4] that are trained to predict binding assay output directly from sequence. However, while powerful, these methods are not without their limitations. It has been recently shown by our group and others that they perform nearly randomly on some variant effect prediction problems when assessing human common variants [5, 6]. Without an underlying mechanistic model, this surprising lack of predictive power cannot be easily analyzed. Therefore, quantifying variant effects from biophysical models of TF binding remains an important approach. The biophysical perspective enables more limited but interpretable models, and thus can provide a critical link between molecular mechanisms and black-box predictions.

Here, we address this need by introducing a highly efficient tool, *motifDiff*, designed to quantify variant effects in the context of mono- and dinucleotide position weight matrices (PWMs) that model TF–DNA interaction. *motifDiff* makes several contributions distinguishing it from previous efforts in this domain [7–9]. It is highly scalable and able to score millions of variants in minutes. It implements several normalization strategies, including an exact correction mapping motif scores to probabilities, which we demonstrate is critical for favorable performance. It supports dinucleotide as well as mononucleotide PWM models. Finally, we evaluate our method on a variety of datasets quantifying effects of common genetic variants *in vivo*, which establishes robust benchmarks for measuring the predictive value of variant effect calculations.

## Approach overview

### *motifDiff* methodology

*motifDiff* is based on modeling the interaction of TFs with DNA via *TF motifs*. In this approach, *TF motifs*can be viewed as generative models for DNA sequences [10]. Sequences from a TFBS are modeled by a position-specific probability matrix (PSPM), where element *ij* represents the probability of nucleotide *i* at position *j* of the motif/binding site. Combined with a background distribution (e.g. marginal nucleotide frequencies of the PSPM), the PSPM entries are transformed into log-odds by dividing each probability by its background counterpart. This results in a position-specific scoring matrix (PSSM) or PWM. Rahmann *et al.* [10], for example, formalized this approach and provided an efficient algorithm to compute the exact distribution of log odds scores given a PSPM and a background model.

**Figure 1.**
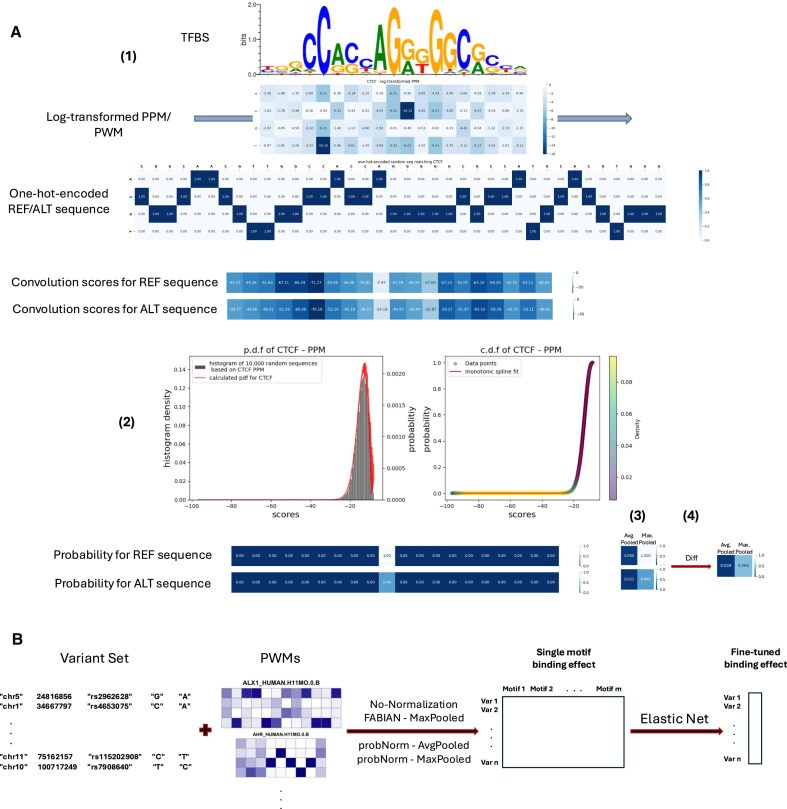
(**A**) Overview of *motifDiff* including four steps: (1) scanning the one-hot-encoded sequence (REF/ALT) by the log-transformed PPM of a TFBS, and calculating the mathematical convolution between them, (2) mapping the calculated convolution scores (on *x*-axis) to probabilities (on *y*-axis) based on the cumulative distribution function (c.d.f) of the motif (normalization), (3) pooling whether to choose the maximum (best match part of the sequence to the motif) or the average (average occupancy of the sequence in the motif distribution) of the mapped scores, and (4) getting the difference between the pooled scores from REF and ALT sequences to see if the best match or average occupancy of the sequence is increased (constructive) or decreased (destructive) by the variant. (**B**) The second phase of analysis for benchmarking, which takes a set of variants along with a set of motif PWMs and calculates the difference in binding of the REF and ALT sequence surrounding each variant. These generated Diffs (single motif binding effects) are then used as features to train an Elastic Net regression model for fine-tuned prediction of the variant effect.

A straightforward approach could be to quantify the effect of a DNA sequence variant by subtracting the log-odds/PWM score of the reference (REF) versus the alternative (ALT) variant, or the respective maxima of variant-overlapping windows the size of the motif length (we will refer to this as No-Normalization). However, this approach forfeits information about the absolute values of the PWM scores of REF and ALT, and of its effect on TF binding. To illustrate, assume the probability of TF binding is related to the PWM score *s* with a monotone function *f*(*s*) with range [0, 1]. Considering Fig. 1A, panel (2), right side, as a hypothetical example of such a curve, clearly PWM score differences between small scores (left side of *x*-axis) have less impact on TF-binding probability than the same differences would have between larger scores (right side of *x*-axis). However, the true *f*(*s*) is not only typically unknown but also context-dependent. In particular, it depends on the concentration of the TF in question and likely on other factors, like the concentration of other TFs and cellular variables, such as post-translational modifications, that may affect TF affinity. To allow for a scalable and general approach, *motifDiff*uses an approach we term probNorm, where we approximate the function *f*(*s*) with *g*(*s*), which is the cumulative distribution function of the PWM derived from the score distribution discussed above.

For this newly proposed probNorm method, we investigate two settings that differ by how the per-position score is summarized (max versus average) across a PWM-scanned and probability-transformed DNA sequence. While taking the maximum binding position for both ALT and REF is a natural choice, the maximum match position can switch between the REF and the ALT, and thus in the max-pool setting, the REF and ALT scores would correspond to different binding events. This is naturally handled by averaging, which considers all possible binding positions. When the scores are normalized to more accurately reflect binding probability, however, the contribution from low-affinity positions is near 0, making the difference between max and average subtle. On the other hand, average pooling on raw scores has no natural interpretation and, as expected, drastically reduces performance. Consequently, we omit average pooling for unnormalized scores from our comparisons.

For *TF motifs*, we use 771 HOCOMOCO human mononucleotide PWMs for all our calculations. Our computational frameworks is flexible and allows us to test different configurations of the variant effect computation. For comparisons, we take raw log-odds difference (“No-Normalization”) as a baseline. We also re-implement the normalization employed in the FABIAN tool [7], to benchmark our approach against. FABIAN primarily relies on a normalization factor unique to each motif, calculated by summing the minimum log-likelihood values across all positions after scaling each column of the PWM up to the maximum value at that column so that the max becomes zero. The maximum match score between a sequence (both REF and ALT) and the motif is then divided by this normalization factor and mapped to a [0, 1] range using min–max normalization. More details about the implementation can be found in the Supplementary Document, Detailed Methods.

We also note that our implementation supports different window sizes (around a genetic variant) for sequence scanning. By default, the window is based on the motif length, so scores from PWM positions that do not overlap the variant are not considered. While this is a natural choice, it could be suboptimal for some downstream applications. For example, if a variant is near but not overlapping a strong TFBS, it may be beneficial to consider the effect of that variant on that specific TF to be 0. We find that motif-based window size was optimal in our evaluations (see Supplementary Fig. S2) and do not consider longer windows further. However, as the correct choice depends on the downstream application, we leave the wider-window option to the user.

### Gold standards for variant effects

The goal for our evaluations is to closely match the typical use case for interpreting noncoding variants in the context of human phenotypes, and we compiled our validation datasets accordingly. Specifically we required that the datasets directly measured the effect of a large collection of naturally occurring variants *in vivo*. The restriction to *in vivo* assays is motivated by the need to model the complexity of TF-binding mechanisms which may include interactions with co-factors and chromatin context. The focus on natural variation is likewise critical as we expect that large effects on functional binding sites are actively selected against [11, 12] and common variants are more likely to influence phenotypes through subtle and quantitative changes.

The studies that satisfy these requirement are primarily of two types. One approach is to perform an assay across a population of individuals and assess the statistical relationship between a variant and a molecular readout via Quantitative Trait Locus (QTL) analysis. Because of linkage disequilibrium (LD), QTL associations are not necessarily causal. Since we do not expect to be able to predict non-causal effects from biophysical models of TF binding, we do not expect *a priori* that perfect predictive performance is possible. Within the caQTL dataset (chromatin accessibility QTL: variants that are statistically associated with chromatin accessibility, see Table 1), it is possible to enrich for causal variants by looking for concordance of variant effect across all populations, and this is the finemapping approach taken by the original study. No finemaping information was available for the bQTL dataset (binding QTL: variants that are statistically associated with TF binding.).

**Table 1.** Datasets used to construct gold-standard for variant effect prediction tasks

Data	Source	Description	No. of variants
**ADASTRA**	Abramov *et al.* [13]	ASB events,	Top 10 TFs: ≥5k
		identified through re-processing human	All: ≈330k
		ChIP-seq read alignments	
**UDACHA**	[14]	Allele-Specific chromatin Accessibility	ATAC: ≈5.6M
		(ASA) by meta-analysis of	DNase: ≈6.5M
		DNase-/ATAC-/FAIRE-Seq read alignments FAIRE: ≈ 45K	
		across a total of 859 cell types	
**bQTL**	[15]	**B**inding **QTL**s for TF and histone modification	H3K4me3: ≈27K
		via pooled ChIP-seq	JunD: ≈157K
			NF-κB: ≈109K
			Pou2f1: ≈102K
			PU1: ≈94K
			Stat1: ≈12K
**caQTL**	[16]	**QTL**s mapped for **c**hromatin	each population: ≈17–34K
		**a**ccessibility in 1000 individuals from 10	Total with consistent
		populations via pooling	Higher Binding Allele: ≈119K

The second approach is to use single genotype sources but leverage heterozygosity by mapping allele-resolved reads to the correct haplotype. This approach is taken in the ADASTRA and UDACHA datasets that quantify allele specific TF binding and open chromatin, respectively. It is important to note that this approach, while not sensitive to long-range LD, still is not able to distinguish between two linked alleles if they often appear in the same sequencing read. Thus, we expect this approach to produce set of alleles that is likely to be causal, but not without error.

While not all of these datasets are measuring TF binding they all measure “local chromatin features” and we make the assumption that these are themselves a function of the local binding of DNA-specific proteins. This assumption is well supported by the observation that local chromatin features can be predicted from local sequence with high accuracy [1, 2, 17]. Importantly, even in the case of a specific TF-binding assay, the assay output cannot be reduced to the direct affinity between the protein and the sequence. Other co-factors and general chromatin context play a role. To account for all of these effects we treat each variant effect prediction method as a feature generator that is then fine-tuned to predict the specific allelic effect task through an elastic-net regression layer trained identically for all inputs (Fig. 1B).

The fine-tuning approach allows us to investigate any dataset that reports variant effects on local chromatin features without needing to know the exact TFs involved. In the few cases where a direct comparison between a single motif score and the variant effect task is possible, we find that the ranking of the variant effect methods is consistent across elastic-net fine-tuning and single motif scores.

Finally, we note that while the predictive power varies considerably both by the variant effect method and the test dataset they are all relatively low. PWM-based methods do not achieve a correlation of >0.5 in any setting and often the actual value is much less. This relatively low predictive performance could be attributed to a number of factors. Potential sources of low predictive power include (i) missing relevant PWMs or other sequence features, (ii) sub-optimal binding approximations, (iii) nonlinear effects not captured by linear fine-tuning, and (iv) noise in the test dataset. The test dataset may indeed contain significant noise due to residual confounding by linked noncausal alleles and insufficient statistical power (low minor allele frequency or few allele resolved reads). In order to distinguish between modeling limitations and noise in the test dataset we compare our variant diff scores to a state of the art deep learning approach, Sei [2]. Using Sei implicitly solves problems 1–3 as it is trained to specifically predict binding, and thus already computes the optimal convolutional feature extractions, binding approximation, and any non-linear effects. As expected, the Sei features achieve the best predictions in many (though not all) evaluations. However, the maximum correlation achieved in any setting is improved only to 0.65 indicating that the low performance is mainly a function of dataset noise rather than modeling limitations.

## Results

### *motifDiff* with probNorm is an effective approach for quantifying noncoding variant effects

We demonstrate our evaluation approach using the ADASTRA dataset, which computes allele-specific binding (ASB) for TFs from allele-resolved reads. This dataset is significant because it directly measures TF binding (unlike other chromatin features) and is relatively well predicted. Our focus is on the 10 variant sets with the largest number of reported variants affecting their target TF (Table 1).

**Figure 2.**
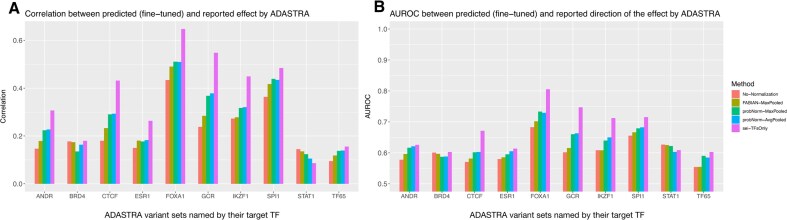
Comparing the performance in fine-tuned predictions by Elastic-Net from single motif predictions calculated by four methods: No-Normalization (raw log-odds diff), FABIAN, probNorm-AvgPooled, and probNorm-MaxPooled for variants in the ADASTRA dataset [13] separated by their target TF. The purple bar corresponds to the fine-tuned predictions from single motif variant effect features produced by the deep learning model Sei [2] and is included for reference as an upper bound on predictive performance. Panel (**A**) shows correlation between the true and predicted values from the regression task, and panel (**B**) shows AUROC between the true and predicted direction of effect from the classification task.

We assess the holdout correlation for a linear regression task predicting a *z*-score like value accounting for both the direction of the variant effect and the significance of the effect size, as −log_10_(*P-*value)*sign(effect), (for more details see Supplementary Document: Evaluation, Data—TF binding effects) (Fig. 2A). Since the quantification of effect size depends on statistical power, which varies by variant, we also report the performance of the binary prediction task for the direction of the effect (Fig. 2B).

**Figure 3.**
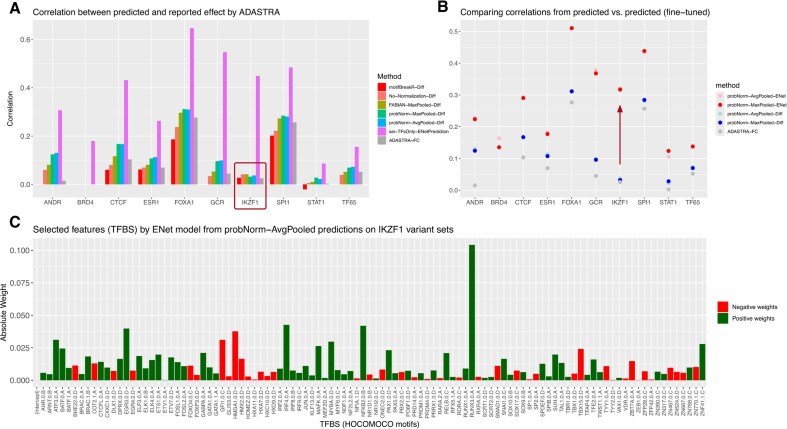
(**A**) Correlation between the *z*-score like values from ADASTRA and the single motif binding effects calculated by the four previously introduced methods, plus motifbreakR [8]. In each variant set, only the column corresponding to the motif for the target TF is extracted to calculate its correlation with the *z*-score like value. The correlation between the logFC reported by ADASTRA and the *z*-score like value is also shown in gray. The correlations from Sei fine-tuned predictions are included for reference. (**B**) The gap between single-motif (referred to as Diff) and fine-tuned (referred to as ENet) predictions. (**C**) single-motif effects as features that are selected in the variant set targeting the binding of IKZF1 by the elastic-net model and their weights on *y*-axis. Note that IKZF1 motif itself is not selected, which explains the large performance gain after fine-tuning.

We emphasize that these 10 TFs correspond to evaluation datasets—that is, groups of variants labeled by the protein that is the target of the original ChIP-seq experiment—not to a restriction on the features used in modeling. For each variant set, we compute predicted binding effects across all 771 HOCOMOCO motifs, producing a matrix of variant-by-motif scores. These are used as input features for elastic-net regression. This design reflects the well-established observation that TF binding is frequently influenced by motifs of additional factors [18]. We note that the predictive value of individual motif features, without fine-tuning, is assessed in Fig. 3. As shown, performance is generally lower than that of the full model, and in some cases do barely outperform baseline, underscoring the importance of incorporating multiple motifs and using regression to capture their combined effects.

As expected, we find that the variant effect features produced by the deep learning method, Sei, perform best on most ADASTRA tasks, establishing a valuable performance benchmark. Among the methods that directly use PWMs, we find that the un-normalized score difference is inferior in 8 out of 10 tasks, while the PDF normalized method we propose performs best in 8 out of 10 tasks. We observe a consistent trend where for tasks that can be well predicted (as measured by Sei’s performance), the sequence of performance is un-normalized ≤ FABIAN ≤ ProbNorm ≤ Sei indicating that increasingly sophisticated methods perform better. For the two tasks in which Sei does not emerge as the top performing method (BRD4 and STAT1), we also do not observe a consistent trend among PWM methods. In these cases, it is likely that the prediction problem is not well specified as even the comprehensive Sei output does not appear to have highly informative features. We also find that the AUC performance for the sign of the variant effect tracks with the correlation performance, indicating that improvement in variant effect prediction is not concentrated in large effects.

Since the ADASTRA dataset is derived from ChIP-seq data of specific TFs, it enables the direct evaluation of single-motif variant effects without the need for fine-tuning. To compare the effects of the variants on single-motif binding against ground truth, each variant set targeting a TF was matched to its corresponding motif using the symbol name. This matching process is streamlined by the fact that the ADASTRA dataset and the HOCOMOCO database, which provides PWMs, are both products of the same research group and have consistent nomenclature. Notably, when matched PWMs are available, the ADASTRA dataset offers its own PWM-based difference score (as the logFC between ALT and REF), which we include in our comparison (Fig. 3A, gray bars). We find that all of the normalization schemes implemented in our tool improve upon the ADASTRA reported diff score; un-normalized log-odds were typically though not always worse, and probNorm was best in all cases with overall good performance (exclusive of BRD4, where the corresponding motif is not available, and IKZF1).

On this single-motif score task, we also compare the performance of another widely used motif scoring tool motifbreakR [8]. This tools was used recently in several high impact studies [19, 20]. Given that this tool performed below probNorm on variant effects and was 180–400 times slower (see timing section below), we omit it from further comparisons.

Our findings indicate that, compared to elastic-net fine-tuning, using the matched motif score directly overall reduces performance, and in some cases dramatically (e.g. IKZF1, BRD4, and STAT1), suggesting that for these TFs, the PWM may not accurately represent binding affinity, or that binding partners and chromatin context play a crucial role. To understand the significant discrepancy between the elastic-net fine-tuned predictions and the single-motif score, we analyzed the features selected by the elastic-net model on the variants targeting IKZF1, see Fig. 3C. Our analysis revealed that a diverse set of TF features is necessary for optimal prediction of IKZF1 ASB, with the largest contribution coming from RUNX1, while the IKZF1 motif feature was not selected. This finding could be attributed to several factors. Both the PWMs and the variant effects are constructed through a computational processing of ChIP-seq data. However, PWMs are constructed from relatively few well supported binding events while variant effects are evaluated everywhere, where there is sufficient haplotype resolved reads and are likely enriched for weaker binding sites or binding through co-complexes.

It is also important to consider the possibility that this observation may be influenced by bias in the alleles themselves. The alleles analyzed by ADASTRA are not random genetic variations but correspond to those naturally occurring in cell lines. Consequently, it is conceivable that alleles capable of directly altering IKZF1 binding might be negatively selected against, leaving only alleles with weaker effects that depend on interactions to be observed. This scenario aligns precisely with our research focus, as we aim to explore the impact of common variants and effects measured *in vivo*.

**Figure 4.**
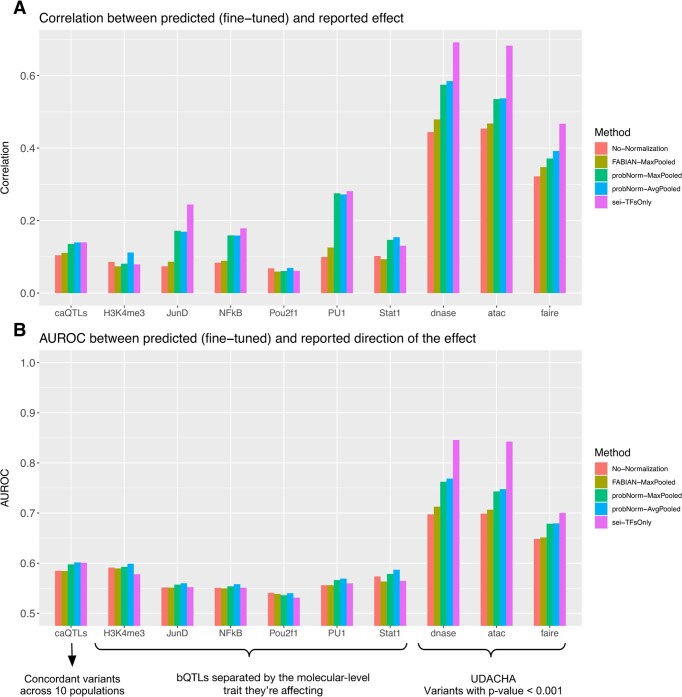
(**A**) Correlation and (**B**) AUROC from the fine-tuned predictions by elastic-net on caQTLs, bQTLs and UDACHA variant set. Only variants with concordant effect across population were considered in caQTLs dataset. In bQTLs dataset, variants are separated by their target TF and Histone Modification. The variants in UDACHA datasets are separated by the experiment and here were filtered to only include the ones with *P*-value ≤ 0.001.

Performance on the other three gold-standard datasets is depicted in Fig. 4. We observe that the pattern of performance is similar to that of ADASTRA, with the probNorm method (represented in green and blue) outperforming other methods in the PWM class on average. Additionally, we note poor performance across all methods on QTL datasets (binding bQTL or chromatin accessibility caQTL). In many instances, Sei’s performance on QTL prediction does not significantly exceed that of PWM-based methods. These results are likely due to the QTLs being a relatively smaller and noisier dataset, which makes the fine-tuning task more challenging. Given that Sei produces more features (≈4000 versus 771 for HOCOMOCO), the difficulty of learning the fine-tuning model is increased. Since there is no principled method to subsample Sei features to match the HOCOMOCO input dimension, a natural choice is to perform Principal Component Analysis (PCA) to reduce Sei's dimensions; however, we found that this further decreased performance.

We note that a potential issue with the QTL datasets is that the variants may not necessarily be causal, due to LD confounding, which naturally limits the theoretically best performance that can be achieved with mechanistic models. We can investigate the contribution of LD confounding using the caQTL dataset where measurements were conducted across 10 different populations. We can enrich for causal variants by considering only those variants with concordant effects, as per the fine-mapping approach adopted by the original study. However, our findings indicate that the performance on the caQTL dataset, even when focusing on concordant variants, does not improve (see Supplementary Fig. S3). This suggests that predicting QTLs is a challenging task for reasons that extend beyond the confounding effects of LD.

Finally, we consider the effect of max versus average pooling. Average-pooling considers all possible binding positions that overlap the variant in question. Max-pooling considers the maximal site only, which has the subtle disadvantage that it may be different in the two versions of the sequence being contrasted.

Average pooling resolves this issue, but at an increased computational cost of multiple queries to the normalizing function. At the same time, changes in the location of the maximal binding score occur rarely. Indeed, in our aggregate performance plots, probNorm-MaxPooled and probNorm-AvgPooled appear similar.

**Figure 5.**
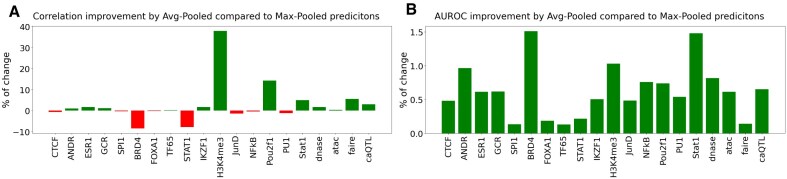
Using average pooling produces up to 40% improvement on effect sizes value prediction in some cases (**A**), and small but consistent improvement on all effect size sign prediction tasks (**B**).

However, expressing the difference as percent improvement, we find that average pooling produces a small but uniform improvement in binary prediction tasks for the variant sign across all variant sets (Fig. 5B). It also shows less consistent but still significant improvement for the quantitative effect size prediction. As the binary prediction task is independent of the magnitude and thus gives more weight to small changes in binding, our results suggest that averaging over all positions is indeed advantageous in this setting.

### Dinucleotide models do not improve variant effect prediction in the majority of cases

PWMs are simplistic models based on the assumption that each base’s contribution to binding is independent. However, interdependencies between nucleotides have been consistently observed in studies [21–24]. Indeed, more sophisticated models have frequently been shown to outperform PWMs in identifying binding sites validated by experiments [25–27]. Attempts to integrate the relationships between nucleotide positions, both adjacent and distant, have led to the development of alternative approaches. Notable among these are the binding energy model (BEM) [28], dinucleotide weight matrices (DWMs) [29], and transcription factor flexible models (TFFMs) [25].

**Figure 6.**
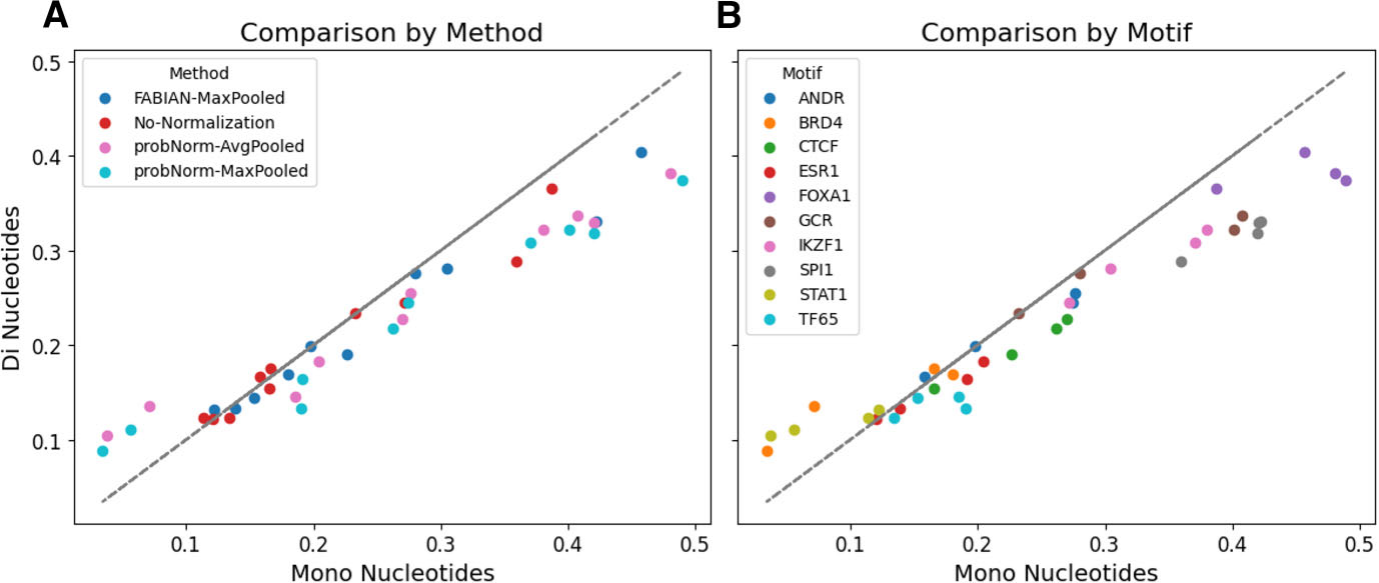
Comparing mono- and dinucleotide models on ADASTRA TF-binding predictions. Each point represents the performance (Pearson correlation) of a specific method on a specific TF-associated variant set. Panel (**A**) colors the points by method, showing variation across the four tested approaches (FABIAN-MaxPooled, No-Normalization, probNorm-AvgPooled, and probNorm-MaxPooled). Panel (**B**) colors the same points by motif, corresponding to the ten ADASTRA variant sets labeled by TF (e.g. FOXA1 and CTCF). Thus, each variant set appears as four points (one per method), and each method appears across ten variant sets.

Our focus is particularly on DWMs because our tool utilizes fast convolutions for scalability, and DWMs can be represented as convolutions with dinucleotide-encoded sequences. Additionally, DWMs are part of the HOCOMOCO database [27] (albeit only for a subset of TFs), enabling a more straightforward comparison. Supplementary Figure S1 shows a descriptive comparison between PWMs and DWMs in probNorm normalization. For this part of our evaluations, we concentrate on the common set of 278 TFs represented as both DWMs and PWMs in the HOCOMOCO database. We observe that dinucleotide models generally underperform compared to mononucleotide models, with the exceptions being STAT1 and BRD4 when using the probNorm method (Fig. 6). These two TFs are exceptions in that their overall performance was low, and the probNorm normalization scheme underperformed relative to No-Normalization. While using dinucleotide models enhances normalized performance, the non-normalized performance (illustrated by red dots in panel A) remains comparable, suggesting that differences are likely due to interactions with normalization rather than inherent qualities of the DWMs.

In conclusion, we find no evidence that DWMs are more effective in capturing variant effects. A potential reason could be the challenge of accurately computing DWM models due to their expanded parameter space. However, the models are likely to improve as data are added and/or reprocessed, and our evaluation approach can serve as a robust benchmarking platform to validate potential improvements to dinucleotide models. Additionally, the complex interplay with normalization indicates that there may be opportunities for enhancing dinucleotide performance through improved normalization strategies.

### *motifDiff* is highly scalable

**Figure 7.**
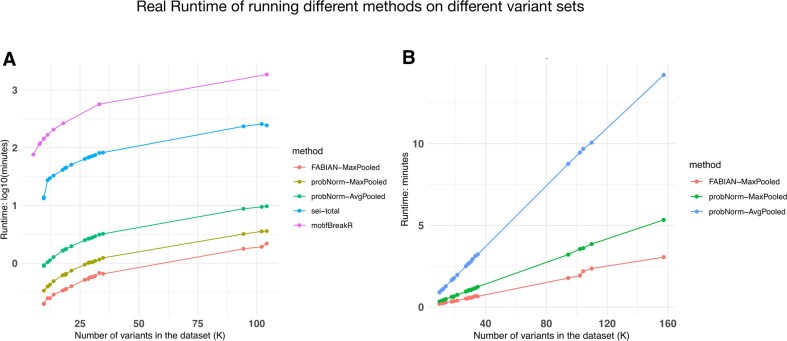
Runtimes for variant effect prediction methods plotted on the log-scale (left panel) and linear scale (right panel, excluding Sei).

One of the driving motivations for our approach is the scalability. We compare the scalability of our approach to another popular motif-based method, motifbreakeR [8], which has been used in several high impact studies [19, 20]. We find that our method is from 180 to 480 times faster (depending on whether max or average mode is considered, respectively) than motifbreakR running single threaded (Fig. 7A). We note that probNorm normalization compared to FABIAN indeed incurs an extra cost due to queries to the empirical CDF function, and this is magnified for the average pooling approach (Fig. 7B). We also note that probNorm is considerably faster than Sei with a 25- to 70-fold speed up depending on the setting.

### *motifDiff* scores provide interpretable signals of TFBS change in evolutionary contexts

Evolutionary analysis is one research area where the importance of incorporating biophysical models is particularly evident. Deep learning models often inherit biases present in the genomes they are trained on. A well-documented example is the tendency of neural networks to rely on lineage-specific repetitive elements [30].

In contrast, the biochemical binding preferences of orthologous TFs are often deeply conserved [31], suggesting that many PWMs remain informative even in highly divergent genomes.

To investigate an evolutionary setting where the changes in TFBS are of interest, we consider the human accelerated regions (HARs) whose functionality has been extensively studied. The current view of HARs is that many of them represent neurodevelopmental enhancers that have rapidly changed their function in the human genome and thus may underlie human specific traits [32].

We analyze the HAR variants reported in Whalen *et al.* [33] from the perspective of TFBS gains and losses using different approaches. The original study did not focus on TFBS level analysis opting to use the higher level Sei sequence classes. Sei sequence classes, which compresses all epigenetic tracks predictions into 40 functional categories, including promoters and different enhancer types. Using Sei functional classes, the authors found that the HARs sequences were equally likely to experience gains and losses [33].

**Figure 8.**
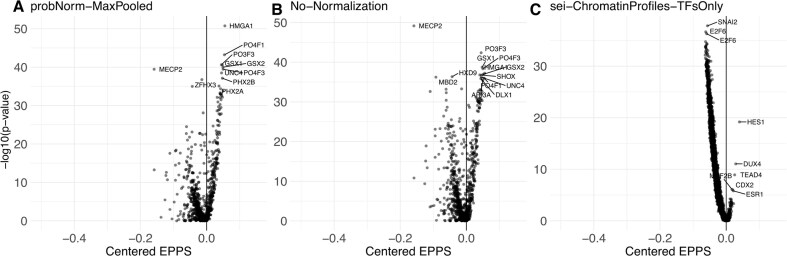
probNorm Diff scores show stronger gain signals compared to Diff scores from Sei. All panels show the positive distribution shift of Diff scores for each TF from Normal distribution on *x*-axis and the significance of the distribution shift on *y*-axis. Since under the null hypothesis the EPPS equals 0.5, we centered it by subtracting 0.5 to clearly indicate binding gains as positive values. (**A**) probNorm Diffs normalized with Max Pooling (**B**) raw log-odd Diffs with no normalization, and (**C**) Sei Diffs only on TF tracks extracted from Chromatin Profiles.

Here, we perform the same analysis from the TF perspective. Specifically, we use different TF scoring methods and compute the Empirical Probability of Positive Shift (EPPS), defined as the proportion of variants with a positive Diff score, leading to increase in binding. Statistical significance was assessed using a Wilcoxon rank-sum test against a normal distribution. We visualized EPPS against −log_10_(FDR-*P-*value). This analysis revealed a overall bias toward gains (Fig. 8A and B), and the TFs associated with gains were strongly enriched for neurodevelopmental roles.

The POU and GSX family TFs play key roles in nervous system development. POU3F3 (also known as BRN1) regulates cortical neuron migration and layer formation during brain development [34]. POU4F1 (BRN3A) and POU4F3 (BRN3C) are critical for the survival and differentiation of sensory neurons, including auditory hair cells, with POU4F3 specifically required for cochlear hair cell maintenance [35, 36]. GSX1 and GSX2 orchestrate regional patterning of the ventral forebrain and spinal cord [37, 38].

We also note that the enrichment in gains is more evident for probNorm when compared to No-Normalization (Fig. 8A and B). At the same time, Sei TF track predictions (Fig. 8C) are highly enriched for losses. The gained TFs also have less direct neuronal relevance, though we note that HES1, DUX4, CDX2, and TEAD4 have critical roles in early development broadly including neurogensis [39–42].

## Discussion

We introduce *motifDiff*, a novel tool designed for scoring variant effects using precomputed mono- and dinucleotide models of TF-binding preferences. We assess its performance across a variety of settings, focusing on ground truth variant effects measured *in vivo* on common human variants—a scenario that closely mirrors the practical application of variant prediction. This contribution is vital, as the accuracy of variant prediction methods can vary significantly depending on the context. For instance, while the complex and high-performing deep learning model Enformer delivers accurate, state-of-the-art predictions on Massively Parallel Reporter Assays (MPRA) data, it demonstrates nearly random accuracy in predicting the direction of common variant expression quantitative trait loci (eQTLs).

Moreover, our tool leverages fast convolutions for sequence scanning, enabling us to conduct comprehensive experiments across different settings. We discover that, as anticipated, raw log-odds differences are not the most effective measure due to the nonlinear relationship between log-odds and binding probabilities. While heuristic methods like those employed by FABIAN offer improvements over raw log-odds, our proposed “probNorm” approach is both more principled and more performant.

As part of our evaluation, we also explore how *motifDiff* can reveal biologically meaningful patterns in an evolutionary context. Using HARs as a case study, we show that *motifDiff* highlights consistent TFBS gains and losses associated with neurodevelopmental regulatory changes. This analysis demonstrates how even simple, interpretable biophysical models can capture evolutionary signatures that may be obscured in more complex frameworks.

In summary, *motifDiff* offers a scalable and refined method for scoring variant effects through biophysical models of TF binding. Although simple in design, this mechanistic framework serves as a foundational element that can be integrated into more complex models, as demonstrated by our application of linear fine-tuning for tasks downstream of TF binding, such as identifying open chromatin regions. Additionally, we envision that this TF-centric framework will offer valuable insights for interpreting both the successes and limitations of powerful black-box models.

## Supplementary Material

gkaf940_Supplemental_File

## Data Availability

All variant sets of the different datasets that we used along with their reported effect sizes and an example script to implement all the analysis to reproduce the final fine-tuned predictions are available for download on Zenodo (https://doi.org/10.5281/zenodo.16697550). *motifDiff* is available as a standalone python package and can be installed by pip install MotifDiff-pkg. All the source code and instructions are also available on github (https://github.com/rezwanhosseini/MotifDiff) and Zenodo (https://doi.org/10.5281/zenodo.15731914). **Reproducing the binding scores** For the first four methods supported by *motifDiff*, probNorm-MaxPooled, probNorm-AvgPooled, FABIAN-MaxPooled, and No-Normalization, follow the instructions provided on README.txt on our github or Zenodo repository or use the example script on our Zenodo repository given above. For the binding scores produced by Sei, we followed the instructions on Sei github (https://github.com/FunctionLab/sei-framework) and extracted the chromatin profiles with the same TFs as the TFs in the HOCOMOCO motif file we used. For the binding scores produced by motifbreakR, we followed the instructions given on https://bioconductor.org/packages/devel/bioc/vignettes/motifbreakR/inst/doc/motifbreakR-vignette.html.
